# Extraction Protocols for Individual Zebrafish's Ventricle Myosin and Skeletal Muscle Actin for *In vitro* Motility Assays

**DOI:** 10.3389/fphys.2017.00367

**Published:** 2017-05-31

**Authors:** Lisa-Mareike Scheid, Cornelia Weber, Nasrin Bopp, Matias Mosqueira, Rainer H. A. Fink

**Affiliations:** Medical Biophysics Unit, Medical Faculty, Institute of Physiology and Pathophysiology, University of HeidelbergHeidelberg, Germany

**Keywords:** muscle myosin II, sliding speed, actin filaments, functional proteins, zebrafish, fluorescence microscopy

## Abstract

The *in vitro* motility assay (IVMA) is a technique that enables the measurement of the interaction between actin and myosin providing a relatively simple model to understand the mechanical muscle function. For actin-myosin IVMA, myosin is immobilized in a measurement chamber, where it converts chemical energy provided by ATP hydrolysis into mechanical energy. The result is the movement of fluorescently labeled actin filaments that can be recorded microscopically and analyzed quantitatively. Resulting sliding speeds and patterns help to characterize the underlying actin-myosin interaction that can be affected by different factors such as mutations or active compounds. Additionally, modulatory actions of the regulatory proteins tropomyosin and troponin in the presence of calcium on actin-myosin interaction can be studied with the IVMA. Zebrafish is considered a suitable model organism for cardiovascular and skeletal muscle research. In this context, straightforward protocols for the isolation and use of zebrafish muscle proteins in the IVMA would provide a useful tool in molecular studies. Currently, there are no protocols available for the mentioned purpose. Therefore, we developed fast and easy protocols for characterization of zebrafish proteins in the IVMA. Our protocols enable the interested researcher to (i) isolate actin from zebrafish skeletal muscle and (ii) extract functionally intact myosin from cardiac and skeletal muscle of individual adult zebrafish. Zebrafish tail muscle actin is isolated after acetone powder preparation, polymerized, and labeled with Rhodamine-Phalloidin. Myosin from ventricles of adult zebrafish is extracted directly into IVMA flow-cells. The same extraction protocol is applicable for comparably small tissue pieces as from zebrafish tail, mouse and frog muscle. After addition of the fluorescently labeled F-actin from zebrafish—or other origin—and ATP, sliding movement can be visualized using a fluorescence microscope and an intensified CCD camera. Taken together, we introduce a method for functional analysis in zebrafish cardiac and skeletal muscle research to study mutations at the molecular level of thick or thin filament proteins. Additionally, preliminary data indicate the usefulness of the presented method to perform the IVMA with myosin extracted from muscles of other animal models.

## Introduction

Since its development in 1986 (Kron and Spudich, 1986), the actin-myosin IVMA has become a valuable and powerful tool to understand muscle function at the molecular level of actin-myosin interaction. The main advantage of the IVMA is to study the molecular motor function under controlled experimental conditions. The method has been continuously improved and is nowadays used in a broad range of applications in muscle research. Only recently, the IVMA was used to analyze the total force of cardiomyopathy related myosin mutants and the effect of a myosin-specific drug on the actin-myosin interaction (Aksel et al., 2015). Additionally, the IVMA has been made commercially available as a ready-to-use kit (CLS Cell Lines Service GmbH, Eppelheim, Germany), enabling the researcher to answer important questions regarding muscle actin-myosin interaction. In this kit, like in most IVMA protocols, a functional part of myosin is immobilized in an assay chamber. Thus, these protocols depend on previously extracted and purified myosin or its functional parts like heavy meromyosin (HMM; myosin head, lever arm with light chain binding sites, and neck) or the smaller subfragment 1 (S1; myosin head and lever arm) (Toyoshima et al., 1987). The isolation protocols for myosins are very time-consuming as it usually takes two days from tissue dissection to isolated protein (e.g., Margossian and Lowey, 1982; Sata et al., 1993) and an additional day for HMM preparation. Once the isolated myosin or a functional part is immobilized in the assay chamber, labeled actin filaments and ATP are added. Thus, the IVMA enables the measurement of the actin-myosin cross-bridge cycle and part of its kinetics. The major parameter measured in the IVMA is the sliding velocity of actin filaments, which is related to unloaded shortening velocity of muscle fibers (Homsher et al., 1992). Modification of the IVMA protein content and buffer composition allows quantification of calcium regulated actin-myosin interactions. In general, this biophysical technique enables a better understanding of the relationship between structure and mechanical properties of actin-myosin interaction and muscle function (Warshaw, 1996). Although basic mechanisms of this interaction are conserved throughout tissues and species, the IVMA with actin and myosin of different origin was and still is required for an in-depth understanding of the effects of mutations or drug effects on actin-myosin interaction.

The zebrafish (*Danio rerio*) is increasingly important as a model organism for research on skeletal and cardiac muscle function under normal and pathological conditions (Dooley and Zon, 2000; Bassett and Currie, 2003; Lin, 2012). However, there is no functional protocol available that could be efficiently used for IVMA with zebrafish muscle proteins. Moreover, the available protocols for myosin isolation from mammalian muscle tissue (Margossian and Lowey, 1982; Canepari et al., 1999; Höök et al., 1999; Thedinga et al., 1999) as well as a protocol for myosin extraction used in carp muscle tissue (Hwang et al., 1991) were not optimal to isolate zebrafish myosin from tail muscles. However, in a first approach we were successful in isolating functional zebrafish skeletal muscle myosin following the protocol published by Elangovan et al. (2012). One general characteristic of the protocols mentioned above are the multiple steps necessary in order to obtain functional myosin, including several polymerization-depolymerization cycles (Margossian and Lowey, 1982; Elangovan et al., 2012). Although a few micrograms of functional myosin are enough for one IVMA experiment (~20–25 μg zebrafish tail muscle myosin per flow-cell), a large amount of original material is needed to compensate for protein loss and to yield a minimum concentration necessary for a series of IVMA experiments. Protein loss is mainly due to the long time needed in these protocols leading to an increase in degraded and non-functional myosin. To compensate for protein loss, it is necessary to increase the tissue amount and thus number of individual animals; Sata et al. used up to 10 rat ventricles for myosin isolations in sufficient concentrations (Sata et al., 1993), and we had to pool a minimum of four zebrafish tail muscles (average weight of 150 mg) to obtain 5 mg of myosin. However, an issue is introduced in terms of statistical analysis, as using pooled samples leads to a reduction of the individual variability. To compensate for this issue, it is necessary to increase the number of repeated measurements with pooled samples and therefore the total number of animals used per experiment. This problem is further enhanced when only a small amount of the tissue of interest is available per animal, such as the ventricle of a zebrafish.

Considering (i) the problems mentioned above, (ii) several approaches that have been published enabling studies of myosin from very small samples like single muscle fibers or even pieces of those (Canepari et al., 1999; Höök et al., 1999; Thedinga et al., 1999) and (iii) the study showing extraction of myosin directly into IVMA flow-cells (Höök et al., 1999) our goal was to develop a method to analyze myosin from individual zebrafish ventricles. To achieve this aim, we combined the protocols of Iorga, Höök and Elangovan (Höök et al., 1999; Iorga et al., 2011; Elangovan et al., 2012) to establish a protocol for myosin extraction directly into the flow-cell. Zebrafish hearts were treated prior to extraction as described previously (Iorga et al., 2011), and could thus be used for myosin extraction within less than two hours after dissection. This step reduced the preparation time considerably as compared to the 24 h freeze-drying procedure (Höök et al., 1999) and ensured the use of fresh tissue samples. In contrast to the buffer from Höök et al. (1999) the extraction buffer used for frog myosin analysis (Elangovan et al., 2012) was successfully applied for myosin extraction from zebrafish tail muscles and allowed us to repeatedly obtain functional ventricular zebrafish myosin. An additional advantage of the direct extraction- besides being fast- is the possibility to study ventricular myosin from individual animals instead of pooling several zebrafish hearts. The extraction protocol is also applicable to other muscles like zebrafish tail muscle.

Further, the direct extraction leads to directed sliding of actin filaments, thus it is even more closely modeling the interaction in intact muscle. The presence not only of the myosin heavy and light chains, but also of the regulatory proteins tropomyosin and troponin in the zebrafish cardiac extracts (Scheid et al., 2016) enables the analysis of calcium regulatory effects on filament sliding.

In parallel to the protocol for zebrafish myosin extraction we provide detailed information on actin isolation from zebrafish skeletal muscle. Taken together, these two protocols enable the interested researcher to study *in vitro* the species-specific actin-myosin interaction. Although muscular actin is expressed with high conservation within a broad range of organisms, the amino acid sequence differs between species. Among others, Asp and Glu residues at the actin N-terminus were identified as binding sites for myosin (Sutoh, 1982; Schröder et al., 1993; Katoh and Morita, 1996). In comparison to the widely used rabbit actin, these residues are conservatively replaced or exchanged in zebrafish actin. These few differences at the relevant positions influence the actin-myosin interaction and thus IVMA results. Although actin is highly abundant in muscle tissue, the small size of zebrafish muscles is the limiting factor in an isolation protocol. Original actin-isolation protocols made use of acetone-powder preparation to break connections within the sarcomeric units. To separate actin from the regulatory proteins still contained in the acetone powder, again, several steps of polymerization and depolymerization are applied (Pardee and Spudich, 1982; Kron et al., 1991). We kept the published protocol, only shortening it by leaving out a second ultracentrifugation step and a consecutive final depolymerization. The yielded zebrafish skeletal muscle actin was functional in the IVMA under calcium-free conditions. Although it is recommended to pool several tail muscles for acetone-powder preparation for high protein yields, it is possible to isolate actin from individual fish, as well.

The usefulness of these protocols was shown in the identification of stress-induced phosphorylation at the ventricular essential myosin light chain (ELC) in zebrafish (Scheid et al., 2016). In this context, the proposed method using zebrafish ventricular and skeletal muscle proteins in the IVMA was used to understand the consequence of the causative mutation at the molecular functional level (Scheid et al., 2016). Taken together, this new protocol is optimized to study cardiac myosin and regulatory protein function as well as skeletal muscle actin from adult zebrafish at the molecular level *in vitro*. Additionally, it is suitable to directly extract myosin from muscle pieces from other species, like mouse skeletal muscle and ventricle or frog ventricle.

## Materials and procedures

### Materials and buffers

A detailed list of materials and equipment can be found in the Supplementary Table 1. Therein, all components are listed in the order of their first appearance in the protocol.

#### Buffer compositions

##### Zebrafish dissection buffer

The composition of the dissection buffer (DB) was very similar to a previously described recipe (Iorga et al., 2011): 10 mM Imidazole, 6 mM Mg-acetate, 5 mM EGTA, 10 mM K-acetate, 47.7 mM Na-creatine phosphate, pH 7.0; one tablet of protease inhibitor cocktail was added to 50 ml DB and 10 ml aliquots were stored at −20°C. Immediately before dissection, 10 ml DB per zebrafish were supplemented with 3 mM Na_2_ATP, 2 mM DTT, 5 mM NaN_3_, and 30 mM BDM. One milliliter thereof was supplemented with 1% TritonX-100; 50 μl/heart were used.

##### Mouse dissection buffer

Mouse muscles were dissected and washed in Ringer-Krebs-Henseleit solution (RKH): 20 mM Hepes, pH 7.4, 150 mM NaCl, 5 mM KCl, 1.2 mM KH_2_PO_4_, 1.2 mM MgCl_2_, 2 mM CaCl_2_.

##### Actin isolation and labeling buffers

Buffer A (BA) for actin isolation contained the following: 2 mM Tris, pH 8.0, 0.2 mM Na_2_ATP, 0.2 mM CaCl_2_, 0.5 mM 2-mercaptoethanol, 0.005% NaN_3_. Furthermore, BA+ was prepared, containing additionally 600 mM KCl, 2 mM MgCl_2_, 1 mM Na_2_ATP.

Labeling buffer (LB) for actin labeling consisted of 3 mM Tris, pH 8.0, 1 mM CaCl_2_, 50 mM KCl, 2 mM MgCl_2_, 1 mM MgATP.

##### Buffers and solutions for myosin extraction and IVMA

*Skinning buffer* (SB) (Wingert et al., 2013) was composed of 20 mM Hepes, pH 7.0, 10 mM EGTA, 4.42 mM Mg(OH)_2_, 8 mM Na_2_ATP, 10 mM Na-creatine phosphate, 10 mM DTT, 50% glycerol.

*Extraction buffer* (EB) was prepared as described previously (Elangovan et al., 2012): 150 mM KPi (see Supplementary Table 1), pH 6.6, 300 mM KCl, 5 mM MgCl_2_, 10 mM Na_4_P_2_O_7_, protease inhibitor, 2.5 mM Na_2_ATP, 10 mM DTT.

*Assay buffer for IVMA* (AB) consisted of 25 mM Hepes, pH 7.4, 25 mM KCl, 1 mM EGTA, 4 mM MgCl_2_, 20 mM DTT; Ca^2+^ containing AB^Ca^ (AB + 10 mM CaCO_3_) was heated under stirring to remove CO_2_. Directly before IVMA performance, 1 ml of each of the following solutions was prepared. Ca^2+^ and ATP concentrations are given as total concentrations. It is recommended to work with stock solutions of the supplements.

- *AB-BSA*: AB + 0.5 mg/ml BSA
- *AB-scav*: AB + 0.5 mg/ml BSA, 3 mg/ml glucose, 0.1 mg/ml glucoseoxidase/0.02 mg/ml catalase
- *AB-ATP* or *AB*^*Ca*^*-ATP*: AB or AB^Ca^ (or a mixture of both to create different [Ca^2+^]) + 0.5 mg/ml BSA, 3 mg/ml glucose, 0.1 mg/ml glucoseoxidase/0.02 mg/ml catalase, 0.5% methylcellulose and up to 10 mM MgATP
- Different combinations of ATP and Ca^2+^ concentrations were generated by mixtures of AB-ATP and AB^Ca^-ATP. To test, for example, 5 mM ATP and 6 mM Ca^2+^, 60% AB^Ca^-5 mM ATP + 40% AB- 5 mM ATP were mixed.

All buffers were degassed on ice in a desiccator for 20 min prior to IVMA performance and stored on ice during the time of usage.

### Animal protocol

All animals used were sacrificed according to the guidelines of the state of Baden-Wuerttemberg; their usage has been approved by the ethics committee of the University of Heidelberg Interfaculty Biomedical Research Facility (permit number mice: T-83/14, permit number zebrafish: T-54/16, permit number frog: T-15/16).

### Stepwise procedures

**Figure 1:**
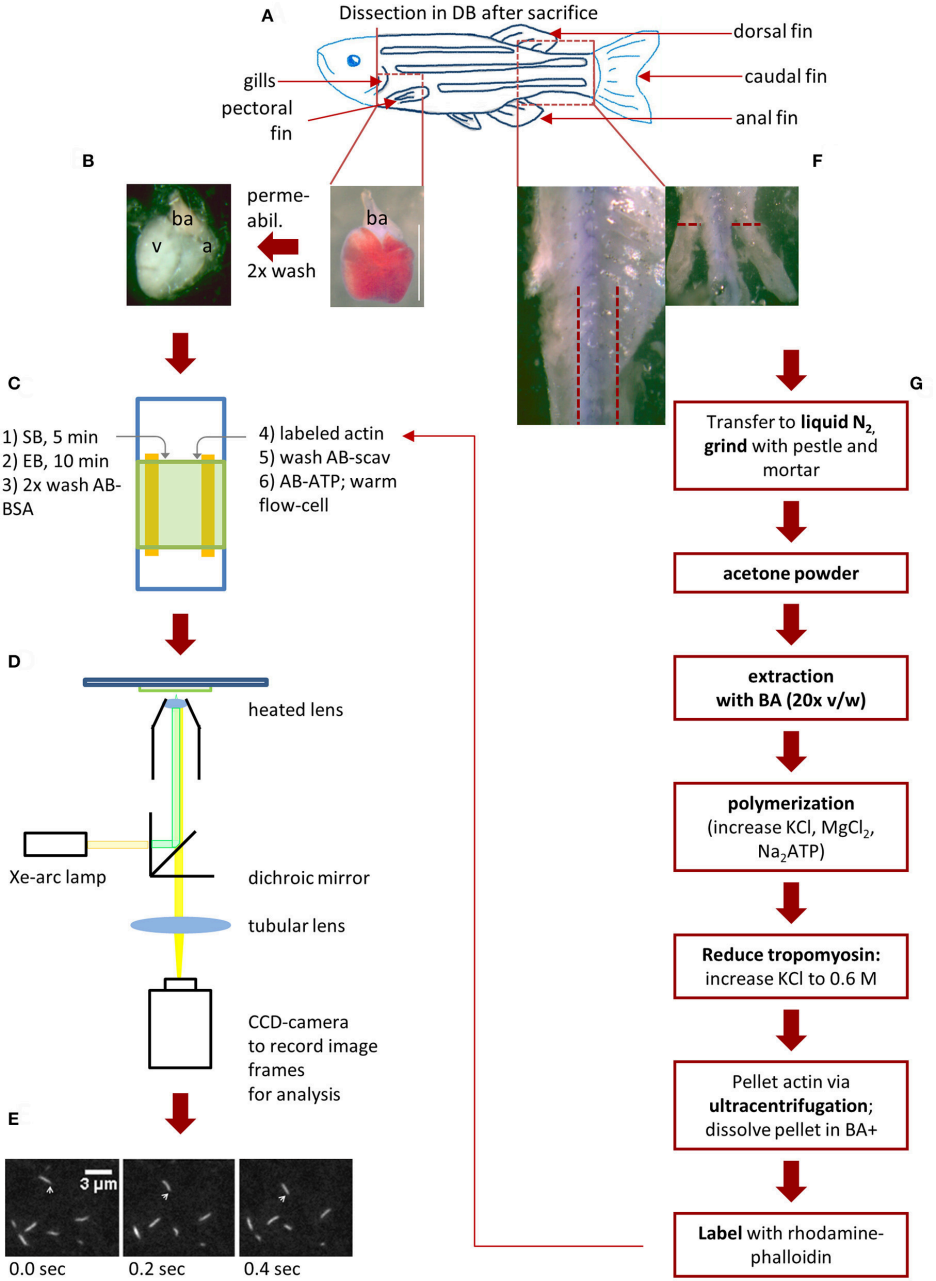
Schematic workflow for zebrafish actin isolation and myosin extraction for IVMA. After the zebrafish (scheme in **A**) was anesthetized with 300 mg/l Tricaine for 10 min, it is decapitated to ensure death. (**B,C**, step 1 and 2): Myosin extraction from zebrafish ventricle. The heart can be exposed after removing the pectoral fin and carefully opening the chest cavity with micro-scissors. By cutting above the *bulbous arteriosus* (*ba*) the heart is set free and can be removed (**B**; scale bar: 1 mm). After permeabilization and washing steps, the *ba* and atrium (a) are removed and the ventricle (v) is transferred into an IVMA flow-cell **(C)**. Consecutively SB (skinning buffer), EB (extraction buffer), AB-BSA are added (steps 1–3 in **C**). Rhodamine-Phalloidin (Rh-Ph) labeled actin is incubated for 2 min in the flow-cell and unbound actin is washed out with AB-scav. After addition of AB-ATP the flow-cell can be pre-warmed (steps 4–6 in **C**). The prewarmed flow-cell is transferred to a microscopic setup enabling detection of Rh-Ph **(D)**. In our case a Xe-arc lamp is used as light-source. A dichroic mirror allows light with the excitatory wavelength (540 nm) to pass toward the flow-cell. Light emitted from the Rh-Ph labeled actin (565 nm) passes through to an intensified CCD-camera. Here, sliding filaments are recorded **(E)** as single image frames (10 fps) and sliding speed can be analyzed as described in **Figure 5**. **(F,G)** Isolation of skeletal muscle actin from zebrafish. After the heart is removed and while it is permeabilized, the tail muscle can be prepared. First, the dorsal, caudal and anal fins are removed as well as the skin in the indicated tail-region. Now the white tail muscle is cut away from the spine **(F)**, transferred to liquid nitrogen and further processed as shown in **(G)**. As the preparation of labeled zebrafish actin takes up to 3 days (including incubation time for labeling) it is usually usable only in the following experiments.

A schematic representation of the workflow from zebrafish dissection to IVMA conduction is given in Figure 1.

#### Zebrafish dissection

Dissection procedures were published previously (Gupta and Mullins, 2010; Iorga et al., 2011; Singleman and Holtzman, 2011; Arnaout et al., 2014; Scheid et al., 2016). Nevertheless, they will be described in some detail to provide information for zebrafish based IVMAs within this protocol. Especially for researchers having no experience in zebrafish handling, we highly recommend the video published by Singleman and Holtzman (2011). The dissection shown within the video for fixated zebrafish is applicable for freshly sacrificed animals in dissection buffer; the dissection protocol described below basically follows that procedure. We used adult wildtype zebrafish at 6–12 months of age. Muscles were either processed directly or snap frozen in liquid nitrogen and stored at −80°C.

##### Heart dissection and preparation

1. Anesthetize zebrafish according to the respective guidelines, i.e., using 300 mg/l Tricaine in tank water on ice for 10 min followed by decapitation to ensure death.
2. After decapitation, pin the fish with its ventral side up into a preparation dish filled with precooled DB. It is easier when the body is slightly turned sideways.
3. Remove the pectoral fin (Figure 1A) and open the chest via a longitudinal incision along the sternum, a horizontal incision between the two pectoral muscles and—if needed—a horizontal incision between the gills using micro-scissors and a binocular preparation microscope.
4. Once the chest cavity is opened, the heart is seen covered by the silvery pericardium. After removing the thin pericardium, the heart is exposed and can be carefully removed by cutting the artery above the *bulbous arteriosus* (*ba*).
5. Directly transfer the heart into a 0.5 ml reaction tube containing 50 μl DB on ice.
6. Carefully spin down (e.g., using a small benchtop centrifuge) and replace the DB with 50 μl DB + Triton. Permeabilize hearts under soft shaking at 4°C for 1 h. Carefully spin down and wash the hearts twice for 15 min in fresh DB. Keep hearts in DB until usage. They are optimally used for myosin extraction at the same day but can be stored up to 3 days at 8°C. Permeabilized hearts can as well be snap-frozen in liquid nitrogen and stored at −80°C for several months. Nevertheless, we recommend myosin extraction from fresh tissue (i.e., 1–3 days after dissection).

##### Zebrafish skeletal muscle dissection

1. After the heart is removed, pin the fish sideways in the preparation dish; use a binocular microscope for the following steps.
2. With micro-scissors carefully remove caudal, dorsal and anal fins (indicated in Figure 1A) as close as possible to the skin.
3. Use micro-scissors to cut open the skin of the fish along the ventral and dorsal sides. Remove the skin using fine forceps being careful not to remove axial muscle.
4. Use a scalpel or micro-scissors to take off the white skeletal tail muscle between the regions of the pelvic fins to the caudal part. Directly place the tail muscles in liquid nitrogen. For actin isolation directly continue as described in the following section. For myosin extraction, tail muscles can be stored at −80°C till use for up to several years.

#### Zebrafish actin

##### Acetone powder preparation

1. Use a mortar and pestle in presence of liquid nitrogen to grind the muscle pieces. Transfer them into a 15 ml centrifugation tube. It is recommended to pool tail muscles from at least 5 adult zebrafish. Nevertheless, it is possible to isolate actin even from a single tail muscle. To give an example, in our hands 350 mg acetone powder were yielded from 10 adult zebrafish.
2. Add 1 ml ice-cold acetone per dissected zebrafish; incubate for 30 min on ice and vortex every 5 min for 30 s.
3. Centrifuge for 10 min at 4°C with 10,600 × g. Resuspend the pellet in fresh ice-cold acetone (same volume as before) and incubate on ice for 10 min; vortex every 5 min for 30 s. Repeat the centrifugation step. Resuspend the pellet in maximally 2 ml acetone and pour it onto a filter paper for air-drying overnight.
4. After the powder has dried, transfer it into a tube and store it at −20°C where it can be kept for several years.

##### Actin isolation and labeling

Actin extraction from acetone powder is based on previously published methods (Pardee and Spudich, 1982; Kron et al., 1991). Best results are achieved, when all steps are performed at 4–8°C.

**Figure 2:**
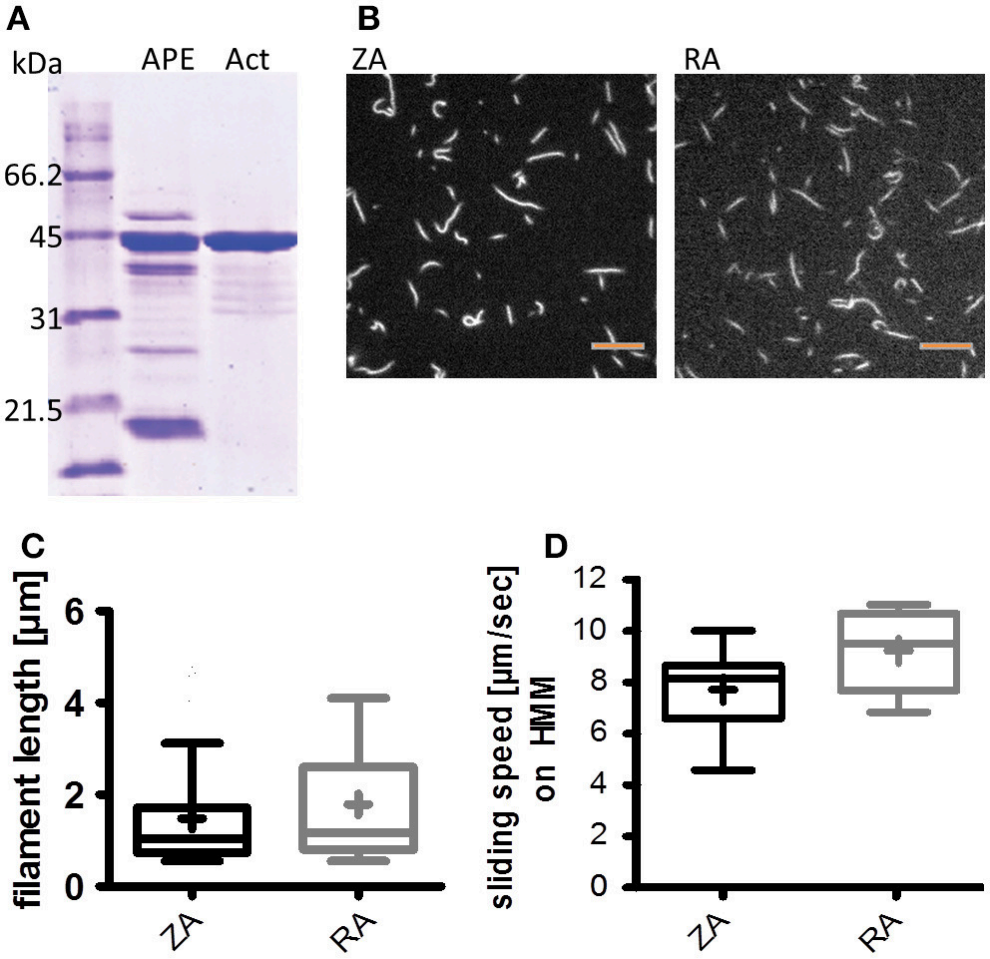
Zebrafish actin isolation from acetone powder has little impurity and can be polymerized; it is functional in the IVMA. **(A)** Coomassie staining of an SDS-PAGE comparing acetone powder extract (APE) and isolated actin (Act; ca. 43 kDa) as described in the protocol section on Zebrafish Actin. **(B,C)** Isolated zebrafish actin can be polymerized and labeled with Rhodamine-Phalloidin. **(B)** Example images of labeled zebrafish actin (ZA, left panel) and rabbit actin (RA, right panel) filaments; scale bars are 5 μm. Filament lengths of rabbit and zebrafish actin were compared in three representative IVMA records and were not different (Mann-Whitney test) **(C)**. **(D)** ZA is propelled by rabbit heavy meromyosin (HMM) and thus usable in IVMA; the median sliding speed is significantly lower (*p* < 0.0001; Mann-Whitney test) than for RA. Boxplots represent the median (line inside the box), 25 percentile (lower limit of the box), 75 percentile (upper limit of the box), and Tukey whiskers. In **(C,D)** the respective means of filament length and sliding speeds are indicated as + inside the box of the boxplots.

1. Add 20x (v/w) BA to acetone powder and extract by gentle shaking on an orbital shaker for 20 min.
2. Centrifuge (15 min, 4°C, 20,800 × g) and keep the supernatant. Repeat the extraction step once with the pellet and pool supernatants after centrifugation.
3. Actin polymerization in the pooled supernatants is induced by adding KCl to 50 mM, MgCl_2_ to 2 mM and addition of 1 mM MgATP. Keep it on ice for at least 1 h; this pre-polymerization step can be done overnight.
4. Stepwise add solid KCl to increase concentration to 600 mM and thus dissolve regulatory proteins. Mix by gentle spinning on a roll mixer for 30 s in between the single additions and for a final 30 min.
5. Pellet pre-polymerized actin via ultracentrifugation (2 h, 4°C, 120,000 × g), overlay the pellet with BA+ and store at 8 °C overnight.
6. When the pellet has dissolved, determine protein concentration (e.g., via Bradford assay). In an exemplary procedure, we yielded 6 mg actin from 350 mg acetone powder. Actin purity can be analyzed with SDS-PAGE analysis after Coomassie staining using freeware like ImageJ (“Analyze—Gels” tools; Supplementary Figure 1) or the commercially available Labimage 1D (Kapelan Bioimaging solutions). The actin protein band (43 kDa) can be identified with the help of a protein molecular weight standard. With the mentioned software tools, protein bands in the lanes on the gel image are plotted as intensity profiles. The area under each intensity peak is determined; the percentage of the actin band related to the sum of all bands represents the purity. Labimage 1D will automatically calculate the percentage for each band.
7. For long-term storage, freeze actin dropwise in liquid nitrogen after addition of 20% sucrose and keep at −80°C. In our hands, zebrafish actin stored this way was still functional after four years, so far.
8. Prepare Rhodamine-Phalloidin (Rh-Ph) for actin labeling according to the manufacturer. For the Rh-Ph used in this protocol, the manufacturer provides 300 U Rh-Ph and recommends to dissolve it in methanol at 200 U/ml (is equivalent to 6.6 μM). 1 U is defined by the manufacturer as “the amount of material used to stain one microscope slide of fixed cells” (https://tools.thermofisher.com/content/sfs/manuals/mp00354.pdf). For each labeling approach prepare 10 μl dissolved Rh-Ph, e.g., in 0.5 ml reaction tubes. Keep the open tubes for ~15 min in the dark to let methanol evaporate.
9. For actin labeling, dilute the concentrated actin to 200 μg/ml in LB and keep on ice. Add 10 μl of actin in LB to each tube with Rh-Ph, resulting in 1 U Rh-Ph per μg actin. We usually label 3 portions per approach. Store the labeling vials in the dark at 8°C for at least 48 h.
10. Similar labeling processes can be performed with actin from different species and tissue origin. For example, rabbit skeletal muscle actin protocols are published elsewhere (Pardee and Spudich, 1982; Kron et al., 1991).
11. Store labeled zebrafish actin at 8°C under exclusion of light for up to 4 months (maximum tested so far). We found no changes in sliding speed in IVMA during this period.
12. Directly before IVMA conduction dilute labeled zebrafish actin in AB-BSA starting with 1:50; higher dilution up to 1:300 may be needed. Actin labeling and density can be determined in a standard IVMA making use of immobilized HMM. A density as shown in Figure 2B would be optimal (i.e., 50–70 filaments in the shown 30 × 30 μm area).

#### Mouse dissection

As the main focus of the presented protocol is the use of zebrafish muscle to extract functional proteins for the IVMA, the mouse protocol is presented less extensively here. Mice (15–25 weeks of age) were sacrificed by cervical dislocation. After disinfection with ethanol, skin was removed to expose the breast and leg muscles. Complete legs were transferred into RKH for further dissection of the fast *extensor digitorum longus* muscles (EDL) as shown previously (Oishi et al., 2011; Moorwood et al., 2013). The heart was transferred to and washed in RKH. Ventricles were dissected and, as well as EDL, snap frozen in liquid nitrogen and stored at −80°C until use.

#### Extraction of myosin into IVMA flow-cells

##### Preparation of flow-cell components

1. Clean microscope slides and 22 × 22 mm cover slides by sonication in isopropanol; air-dry microscope slides and wipe cover slides dry using fuzz-free paper tissue.
2. Dissolve nitrocellulose in acetone to a 3% solution (w/w). Dilute in iso-amyloacetate to 0.2% solution (v/v) and dip cover slides into the solution. Air-dry the coated slides overnight under protection from dust.
3. Glue two thin strips of double-sided adhesive tape with ~2.5 cm length onto a cleaned microscope slide.

##### Myosin extraction

During myosin extraction and IVMA performance, 25 μl of respective solution are used per step. Pipet above the rim of the cover slide, thus the solution will flow by capillary forces and gravity through the chamber. As SB and AB-ATP are viscous, these solutions will flow very slowly into and through the assay chamber. Using a paper tissue to draw at the lower rim of the assay chamber will speed up the respective assay steps. Figures 1B,C schematically represents these assay steps.

1. Transfer the zebrafish heart onto a microscope slide with a drop of DB; carefully remove atrium and *ba* to have only the ventricle left. The size of an adult zebrafish ventricle is ~1.5 × 1–1.5 mm. For extraction from bigger tissue, cut an accordingly small piece of tissue.
2. Place the piece of tissue or zebrafish ventricle (Figure 1B) within the upper third in between the adhesive tape strips on the microscope slide. Using a scalpel cut into the tissue a few times (i.e., five). Carefully fix the cover slide with the nitrocellulose facing down onto the tissue, thus generating the flow-cell for IVMA conduction. Press the cover slide firmly onto the adhesive tape to ensure a leakproof assay chamber.
3. Bring the flow-cell in a tilted position (~45°), pipet SB into the chamber; incubate in horizontal position for 5 min above ice.
4. Wash once with EB and incubate the flow-cell with EB in a tilted (~20°) position for 10 min above ice. Directly continue as described in the following section (IVMA Conduction).
5. For qualitative analysis of the extraction, perform the same protocol with the skinning and extraction steps in a reaction tube followed by SDS-PAGE and Coomassie staining or western blot analysis.

#### IVMA conduction

Directly after myosin extraction, perform IVMA as follows (Figure 1C):

1. Wash the flow-cell twice with AB-BSA.
2. Add labeled and diluted zebrafish or rabbit actin and allow binding to myosin for 2 min; examine the binding of actin filaments microscopically (excitation: 540 nm; emission: 565 nm; 63x or 100x oil immersion lenses).
3. Unbound actin filaments are washed out with AB-scav and the cross-bridge cycling and thus actin filament sliding is induced by the addition of AB-ATP.
4. Pre-warm the flow-cell on a heating plate to ~30°C before mounting it to the microscope for data recording (this step is optional but highly recommended). TIP: since it can take a while to detect sliding filaments; use the extracted tissue visible in bright field for orientation and focusing. Sliding filaments can be usually found downstream of the tissue and directly at and close to the rim of the tissue.
5. Flow-cells can be washed with AB-scav and AB-ATP with different Ca^2+^ or ATP concentrations can be tested. We were able to test up to 4 buffer compositions on one myosin extract. Flow-cells after myosin extraction can be used up to 60 min if they do not fall dry and are stored on ice between measurements.

#### Data recording and analysis

For recordings we used the following setup (Figures 1 D,E):

1. Olympus IX 70 inverted microscope equipped with a Xe-lightsource and the filters for Rh-Ph detection. A100x oil-immersion objective lens was used, equipped with a self-built heating device to provide 30°C in the flow-cell during recording (this is optional, as sliding can already be observed at room temperature).
2. Sliding was recorded with an intensified CCD camera and a camera application software (in our case pco.CamWare). Several records of sliding actin filaments at different spots per flow-cell were taken; we recommend a length of 10 s and a framerate of 10 fps.
3. Records can be analyzed depending on the respective questions and the quality of recordings by using manual tracking approaches [e.g. the plugin Manual Tracking in ImageJ (Schneider et al., 2012)] or available automatic analysis software (e.g., FAST by Aksel et al., 2015) as shown in Figure 5. Additionally, we performed analysis of actin sliding speeds using an in-house analysis software that calculates sliding speeds based on gray-value shifts between consecutive image frames; the software making use of the structure-tensor method with high accuracy was based on a previous publication (Uttenweiler et al., 2000). Histograms of signal counts per speed (in μm/sec) are fitted with a Gaussian curve to determine average sliding speed per record.
4. Graphic representation and statistical analysis of the data was performed using Graphpad Prism 5.Ink (GraphPad Software, San Diego California USA, http://www.graphpad.com). For statistical analysis, first D'Agostino & Pearson omnibus normality test was performed. For data not passing, Mann Whitney test was run to identify statistically significant differences between medians of two data sets.

## Anticipated results

The method described here is schematically depicted in Figure 1 from zebrafish dissection to IVMA conduction. It provides a fast option to extract myosin from zebrafish ventricle and other small muscle tissue pieces. Additionally, we describe how to isolate and label zebrafish skeletal muscle actin for IVMA.

Zebrafish skeletal muscle actin (zebrafish actin, ZA) was isolated from acetone powder prepared of zebrafish tail muscles. ZA was checked for impurities on Coomassie stained SDS-PAGE to ensure the use of actin with minimal additional protein content. Different batches of isolated ZA (analyzed with ImageJ as described in the section on Actin Isolation and Labeling) were between 83 and 92% pure (Figure 2A and Supplementary Figure 1), although the original protocol (Pardee and Spudich, 1982; Kron et al., 1991) was shortened to keep protein yields as high as possible. In our hands, 350 mg acetone powder from 10 zebrafish tail muscles yielded 6 mg actin. For further analysis, ZA was compared with actin from rabbit skeletal muscle (rabbit actin, RA) which is widely used in the IVMA. Despite up to 17% additional protein band intensities at molecular weights differing from actin in Coomassie stained SDS-gels (Supplementary Figures 1 D,E), the purified ZA was found to polymerize to similar filament lengths as RA, as visible with Rhodamine-Phalloidin labeling (Figures 2B,C). Labeled ZA was found to be functional in IVMA using an established standard protocol with heavy meromyosin of rabbit origin (HMM) (Figure 2D); no differences in filament length or sliding speeds were found between ZA batches of different purities.

**Figure 3:**
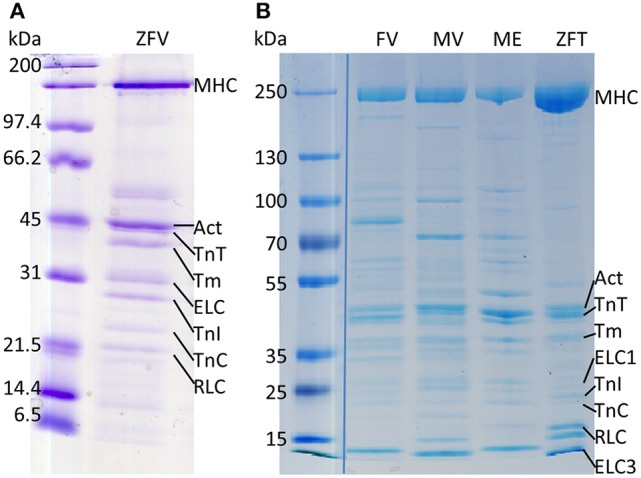
Myosin extracts contain all relevant proteins for (regulated) IVMAs. All used muscle tissues were extracted for SDS-PAGE analysis in a reaction tube using the same protocol as for extraction into IVMA flow-cells (protocol section Myosin Extraction). **(A)** Coomassie staining of a SDS-PAGE with zebrafish ventricular myosin extract (ZFV) of wildtype zebrafish and **(B)** myosin extracted from frog ventricle (FV), mouse ventricle (MV), mouse EDL (ME), and zebrafish tail muscle (ZFT). Myosin heavy chain (MHC), actin (Act), tropomyosin (Tm), troponin T (TnT), troponin I (TnI), troponin C (TnC), ELC and regulatory myosin light chain (RLC) protein bands are indicated. In fast skeletal muscle two ELC isoforms are expressed (indicated as ELC1 and ELC3). Protein identification with western blot analysis for ZFV was published previously (Scheid et al., 2016). The image in **(B)** is a merge of the standard and myosin extracts originally running on the same gel but with several samples in between; merge is indicated by the blue line. The whole gel image can be found in the Supplementary Figure 2.

**Figure 4:**
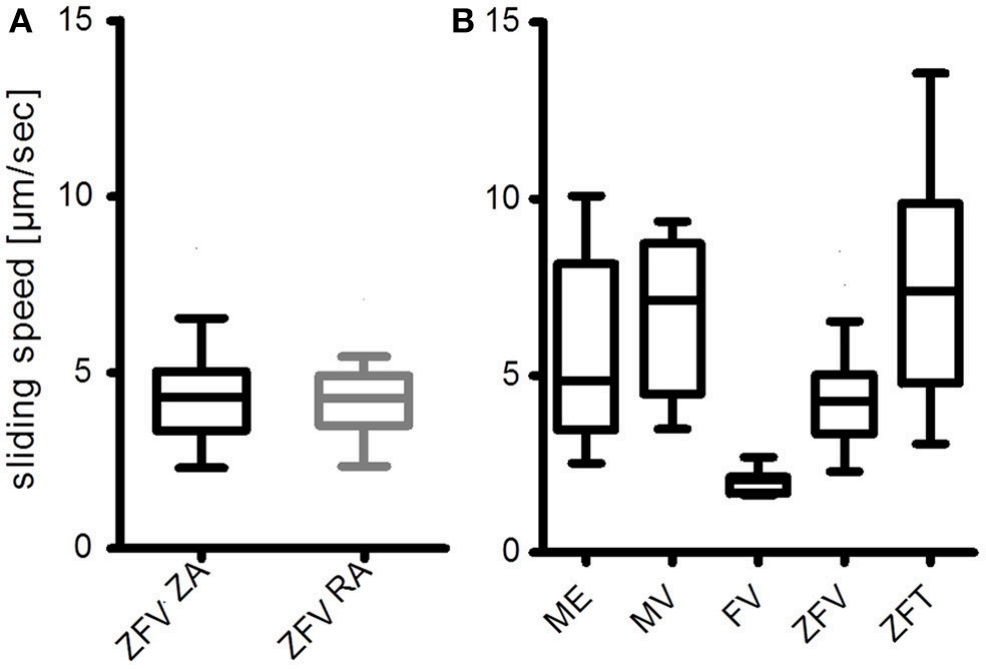
Myosin extracts into IVMA flow-cells are functional. **(A)** Ventricular zebrafish myosin (ZFV) is equally functional with zebrafish actin (ZA) and rabbit actin (RA), as the medians are not different (Mann-Whitney test). **(B)** Preliminary results of myosin extracts from mouse *extensor digitorum longus* (ME), mouse ventricle (MV), frog ventricle (FV), and zebrafish tail muscle (ZFT) are compared to the results with zebrafish ventricle myosin extracts (ZFV) from **(A)**. All myosin extracts were able to propel rabbit actin filaments. Sliding speed distributions where most homogeneous on FV and ZFV. The presented data were analyzed with an in-house software based on gray-value shift determinations between consecutive image frames of normalized records. Average sliding speeds per record were determined by a Gaussian fit of the resulting histograms (Figure 5C). Boxplots represent the median (line inside the box), 25 percentile (lower limit of the box), 75 percentile (upper limit of the box), and Tukey whiskers. As the result of the experiment shown in **(B)** was mainly qualitative, no statistical analysis was performed.

With the described protocol, myosin from single zebrafish ventricles (ZFV) can be extracted directly into IVMA flow-cells. SDS-PAGE and previously published western blot analysis of the extraction confirmed the presence of myosin heavy and light chains in the extracts (Figure 3A and western blot data published in Scheid et al., 2016). Similar protein patterns were found after using the same extraction protocol for ventricular myosin from frog (FV) or mouse (MV) and skeletal muscle myosin from zebrafish tail (ZFT) and mouse EDL (ME) (Figure 3B). Zebrafish ventricular myosin was found to be functional in IVMA and propelled ZA and RA of skeletal muscle origin at the same speeds (Figure 4A). In further experiments, we were able to use the myosin extraction protocol also on pieces of ZFT or ME and MV in combination with RA. For these tissues, sliding actin filaments (RA) were detected and sliding speeds could be analyzed (Figure 4B). Sliding speeds of RA on skeletal muscle extracts (ZFT, ME) were distributed over a higher range as compared to ventricle extractions. Due to the extraction into the flow-cell, formation of “myosin-streets” was observed microscopically, resulting in directed sliding of actin filaments.

**Figure 5:**
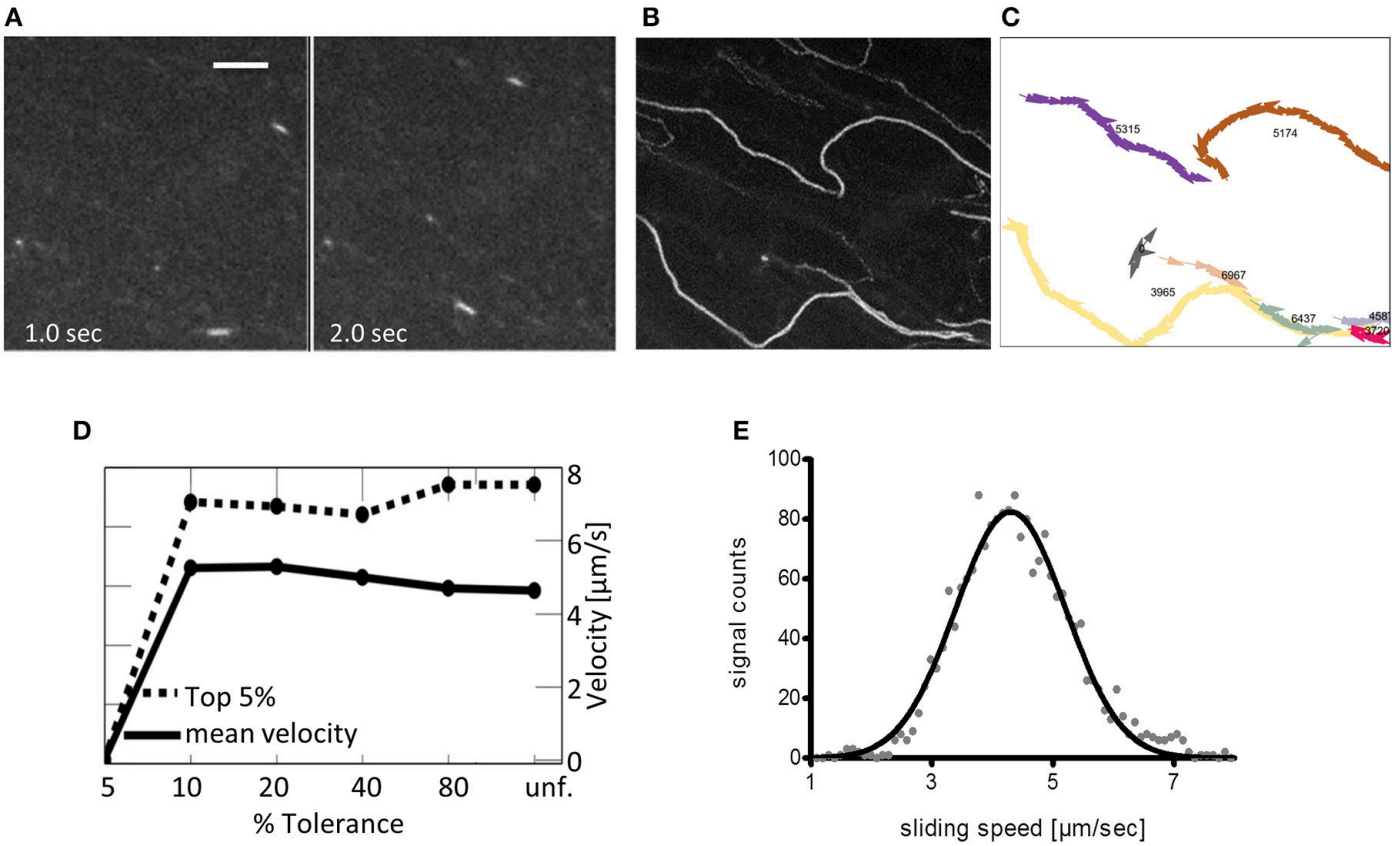
Suggested analysis of a representative IVMA record. Depending on the interest of the researcher and on the quality of the IVMA record, there are different options for the motility analysis. **(A)** Image frames at indicated time points of a representative record with 100 frames in total and a framerate of 10 fps; scale bar is 5 μm. Zebrafish actin is propelled by ventricular zebrafish myosin. The high signal to noise ratio (SNR) allows for automated analysis as shown in the following. **(B)** Z-projection (max. intensity) of the whole record in **(A)** to visualize the overall-tracks of single filaments. **(C)** Single filament tracks automatically calculated by the analysis program FAST (Aksel et al., 2015). Manual tracking is more time consuming, giving similar results in instantaneous speeds based on the frame-to-frame differences in filament-location and can be used for filaments with lower SNR. Mean sliding speeds can be calculated from the data in **(C)**. **(D)** The algorithm of Aksel et al. (2015) calculates both the mean velocity (solid line) and the top 5% velocities (dashed line) based on tracking data. Both mean velocity and top 5% velocity are displayed in relation to % Tolerance, a measure of smoothness of filament sliding (lower values indicate less tolerance to variation in filament velocity, i.e., all filaments with velocity variation above the indicated percentage are removed by the filter; unf. = unfiltered). **(E)** Average sliding speeds per record can also be determined with using gray-value shifts between consecutive image frames and a Gaussian fit of the resulting histogram. The presented analysis was performed with in-house software (Uttenweiler et al., 2000).

To analyze sliding speeds of actin filaments in the myosin extractions, different approaches can be applicable. For cases with a low background and thus high signal-to-noise ratio (SNR) of the sliding filaments (Figure 5A, a maximal intensity based Z-stack of the whole record is shown in Figure 5B), automated tracking based algorithms can be used for analysis. One example is the program FAST that was published recently by Aksel et al. (2015). FAST determines *inter alia* mean velocity and the top 5% sliding velocities of filaments tracked; both are given in dependence on tolerance filtering (Figures 5C,D). For records with a higher background and thus lower SNR, analysis software based on gray-value shift determinations between consecutive image frames of normalized records is recommended (see also the section on Potential Problems, Troubleshooting and Practical Advice, paragraph “*Data analysis*”). With the latter, average sliding speeds per record were determined by a Gaussian fit of the resulting histograms (Figure 5E). The software used in this case was in-house software based on the work of Uttenweiler et al. (2000).

**Figure 6:**
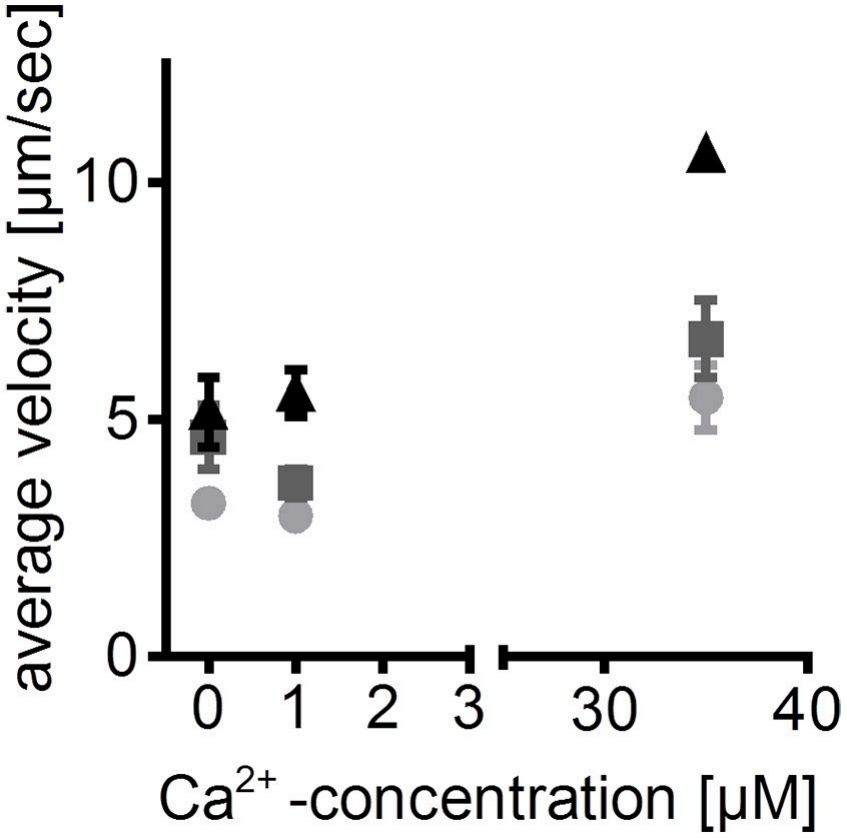
Calcium- and ATP-dependence of zebrafish ventricular myosin- an example of application. Sliding speeds of zebrafish actin filaments on ventricular zebrafish myosin at different ATP concentrations are dependent on free Ca^2+^ concentrations. Thus, reconstitution of labeled actin filaments with regulatory proteins in the flow-cell is assumed. This assumption is based on previously published detection of regulatory proteins in the myosin extracts (Scheid et al., 2016). Average sliding speeds of 5 manually tracked actin filaments ±SEM are depicted; light gray circles: 1 mM ATP; dark gray squares: 5 mM ATP; black triangles: 10 mM ATP. ATP is given in total concentration; free Ca^2+^ concentrations were calculated using “Ca/Mg/ATP/EGTA Calculator” (maxchelator.stanford.edu/CaMgATPEGTA-TS.htm).

The described protocol enables analysis of functional zebrafish myosin in interaction with labeled actin. Besides basic characterization of species-specific actin-myosin interaction, the zebrafish IVMA can be used to analyze molecular effects of mutated muscle proteins and posttranslational modifications (Scheid et al., 2016). In addition to myosin heavy and light chains, the presence of regulatory proteins (tropomyosin and troponin T) in the myosin extracts had been detected (Scheid et al., 2016). Thus, in the present study the extracted ventricular zebrafish myosin was further analyzed regarding the influence of ATP and Ca^2+^ concentration on the actin sliding speed (Figure 6). Increasing concentrations of either of the two components led to increases in average sliding speeds. Therefore, the presented protocol provides a method to qualitatively study regulatory proteins, besides enabling analysis of actin and myosin function. As the amount of regulatory proteins is relatively low and exogenous labeled actin filaments are added, not all actin filaments will be regulated and thus sliding also at 0 μM Ca^2+^ can be observed.

### Potential problems, troubleshooting and practical advice

In this section some potential difficulties and pitfalls are summarized that were or can be encountered.

#### Actin labeling

As mentioned in the section on Actin Isolation and Labeling at point 9, labeled zebrafish actin filaments should be visible microscopically after 48 h incubation of the labeling mix on ice. In a few cases we encountered that after this time only very short (below 0.5 μm) filaments were present on flow-cells in the absence of ATP. Usually, another 48 h of incubation of the labeling mix was sufficient to increase filament length to an average of ~1.5 μm. However, in some cases a prolonged polymerization time might not be enough to increase filament length. Especially when protein impurities in the isolated actin are present, an adaptation of the protein concentration for labeling could result in longer filaments. If polymerization of actin with impurities is slow, an increase of the protein concentration to compensate for the impurities (e.g., in case of 10% impurities, increase total protein concentration also 10%) can be helpful. However, we always yielded good results with the indicated protein concentration, even in cases of impure actin (83% actin band intensity, Supplementary Figures 1D,E). Thus, we did not evaluate the zebrafish-specific critical concentration needed for formation of actin-nuclei as starting points for polymerization (Oosawa and Kasai, 1962; Frieden et al., 1980). This would be an option when no polymerization can be obtained with the slight adaptation of protein concentrations. When polymerized actin filaments with inhomogeneous fluorescent labeling are observed, a too low Rh-Ph concentration can be the cause. An addition of Rh-Ph (e.g., 0.2 U per 10 μl labeling portion) can improve the intensity. Addition of higher Rh-Ph amounts is not recommended as excess Rh-Ph will bind to the endogenous actin present in the extract. Although this will not lead to polymerization of the endogenous actin in the short time of measurement, it does increase the background signal and lower the SNR.

#### Microscopic evaluation

**Figure 7:**
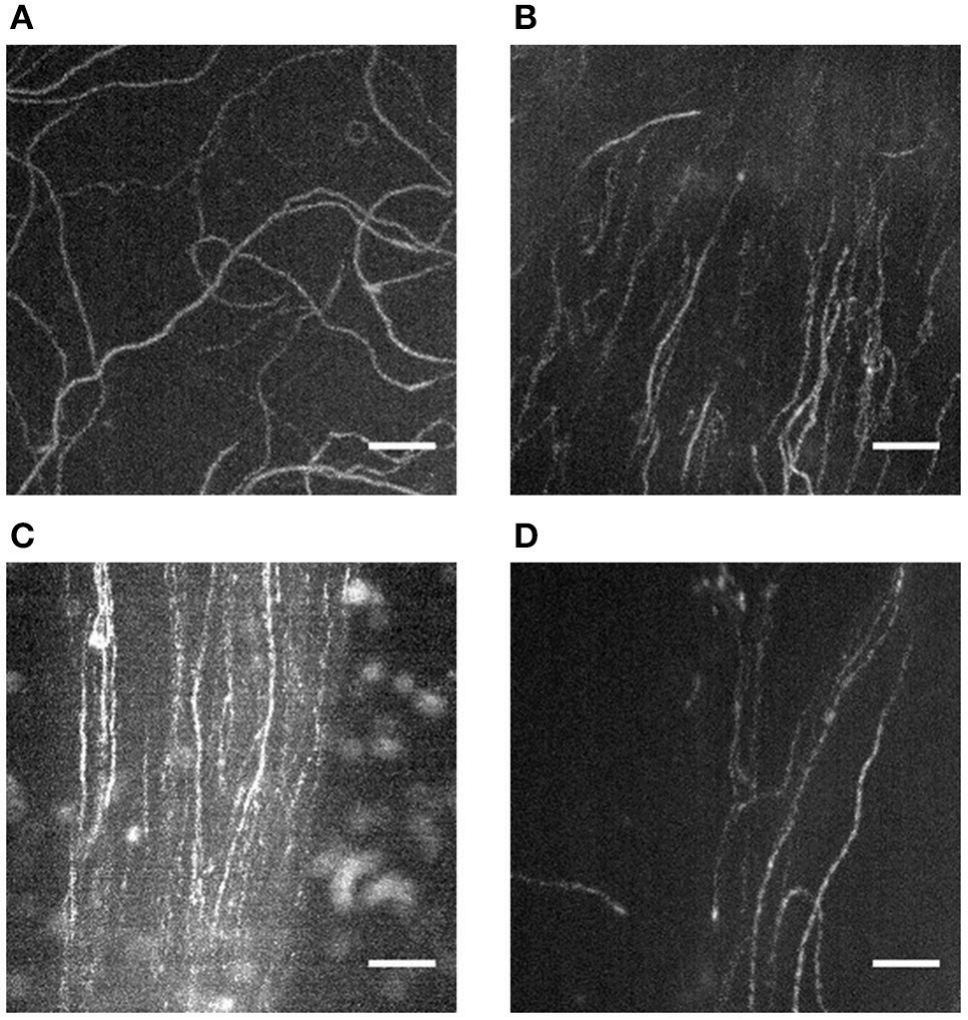
Actin filament sliding on directed paths and tracks of extracted myosin. Z-projections (maximal intensity) of records taken during IVMA *via* ImageJ (Image—Stacks—Z Project) reveal the sliding paths of labeled actin filaments. When pre-isolated myosin or HMM is introduced into the IVMA flow-cell, the resulting actin sliding paths are random **(A)**. When myosin from a tissue piece (in **B–D** ZFT) is extracted, actin slides on distinct and directed tracks. In some areas the track can be broad with a low background signal **(B)**; often distinct tracks **(C)** with a higher background occur. In some cases the tracks are very narrow with only few actin filaments sliding **(D)** and thus need longer time to be detected. Scale bars are 5 μm each.

Although actin filaments can polymerize to lengths of up to 10 μm in the presence of Rh-Ph *in vitro*, their width is very small (i.e., 7 nm). Due to their low fluorescence intensity, the used camera needs to have a very sensitive sensor; usually an intensified CCD (charge coupled device) sensor is recommended. Additionally, the camera should have cooling to reduce thermal noise. Besides the adequate equipment, some practice is needed to find the focus plane with actin filaments in IVMA. Thus, for researchers new to the field of IVMAs, it is recommended to practice the handling of flow-cells, detection and recording of actin filaments in a “standard” IVMA where actin filaments are homogeneously distributed throughout the flow-cell resulting in random actin paths (Figure 7A). There are several publications available on the IVMA (Kron and Spudich, 1986; Toyoshima et al., 1987; Kron et al., 1991; Warshaw, 1996) and a commercially available ready-to use kit (CLS Cell Lines Service GmbH, Eppelheim, Germany). In the flow-cells with extracted tissue, use of the extracted tissue visible in bright field is recommended for orientation and focusing. Sliding filaments can be usually found downstream of the tissue and directly at and close to the rim of the tissue. Actin filaments will usually slide on the traces of myosin that has been washed out of the tissue, as previously described (Höök et al., 1999) and shown in Figures 7B,C. Usually only one track per flow-cell occurs; several records can be taken along the track. In some cases, very dense “streams” of actin with a high background level due to endogenous non-polymerized actin occur that can be difficult to analyze. While taking the IVMA records, areas where actin filaments are clearly visible in these streams should be chosen. Occasionally, these streams of actin sliding on myosin can be very narrow (Figure 7D) and short. Thus, further time for searching might be needed until a representative area in the flow-cell is reached.

#### Data analysis

Possible software tools for data analysis of IVMA records are given in the section on Data Recording and Analysis as well as in the Anticipated Results. A structure-tensor based algorithm provides a robust analysis method. Nevertheless, in some cases automated analysis might not give any results. We observed this especially for the IVMA data with actin sliding on myosin tracks with a low SNR. Here, manual tracking of filaments, ideally after background subtraction, is recommended. This can be done by e.g., using the Manual Tracking plugin of ImageJ. In this case some parameters like time interval and x/y calibration need to be entered manually.

## Discussion

We here present protocols that enable researchers to study myosin function within half a day. The buffer system described has been shown to work for myosin extraction of skeletal and cardiac muscle of both mouse and zebrafish as well as frog ventricle. Small amounts of tissue are sufficient to gain functional insights. Especially in cases of zebrafish ventricle analysis this is an advantage. Extraction of myosin from a single zebrafish ventricle (cross-sectional area ~0.5–1.0 mm^2^) directly into IVMA flow-cells makes it possible to analyze functional cardiac myosin of an individual animal. In some cases, to extract even less than half a ventricle was sufficient for functional analysis. The method provided is also useful for similarly small tissue pieces of mouse skeletal or ventricular muscle, frog ventricle or zebrafish tail muscle. Höök and coworkers were able to even use single myofibers for myosin extraction into IVMA flow-cells (Höök et al., 1999). The protocol published by Höök and coworkers made use of time consuming freeze-drying of the respective tissue. However, when testing the extraction buffer on fresh tissue, we could not extract functional myosin from various types of muscle tissue. The extraction buffer that was working for zebrafish, mouse and frog tissue was as described by Elangovan et al. (2012) and used after a skinning buffer combining glycerol and relaxing components (Wingert et al., 2013). As we could yield functional myosin from such diverse tissue types, the protocol will be likely applicable for an even broader range of tissues. For instance, first functional myosin was extracted from mouse diaphragm (unpublished data). Not only for diaphragm it is of great interest to use small muscle pieces for myosin extraction as different myosin isoforms can be expressed in specific muscle regions. However, there is a drawback in using only such a small amount of tissue for extraction, as the area where functional myosin is immobilized in the flow-cell resulting in actin sliding is relatively small. For developing experience in finding those areas, we suggest using tissue pieces at the size of a whole zebrafish ventricle. In general, the advantages of the direct extraction outweigh the drawbacks. In comparison to other possible methods that prepare myosin by isolation protocols outside the flow-cell (e.g., Margossian and Lowey, 1982), there are several advantages of our direct extraction method: (i) myosin can be extracted within a few minutes from dissected tissue, (ii) due to only a few preparation steps, the loss of functional protein is minimal, (iii) actin sliding mainly occurs following coherent paths due to a more directed orientation of the immobilized myosin (Figure 7), and (iv) in general, myosin can be extracted from ventricles of individual zebrafish and very small muscle pieces. The use of tissue from individual animals is also of relevance in statistical analysis. Isolation of myosin from such small tissue pieces would rely on pooled samples in the most protocols. The resulting presence of myosin from several individuals does introduce variation into the measured samples. Myosin extraction from individual zebrafish ventricles prevents this issue. As stated in advantage (iii), direct myosin extraction leads to smoother sliding of actin filaments, reducing the turns that occur in the sliding paths. Such turns are often observed in IVMAs making use of pre-isolated myosin introduced into the flow-cell (Figure 7). In contrast, smooth sliding is visualized in Figures 5D, 7B–D, where all mean and top 5% sliding velocities revealed <10% variation in sliding velocities of analyzed filaments. Fast myosin extraction [advantage (i)] was especially evident when effects of ELC-phosphorylation were studied with myosin extractions from zebrafish ventricles (Scheid et al., 2016).

During this study, the presence of myosin heavy and light chains and regulatory proteins in myosin extracts from zebrafish ventricles was demonstrated. Together with the isolation protocol for zebrafish actin, this allows researchers to study mutations not only on functional proteins of myosin and actin filaments of zebrafish muscle tissues but also those proteins responsible for Ca^2+^-regulation. Nevertheless, there is a limitation in the latter, as not all actin filaments are present in a regulated form and thus already slide at 0 μM Ca^2+^ (Figure 6). When explicitly Ca^2+^-regulation is studied, the protocol needs further optimization to increase the percentage of regulated actin filaments, i.e., on the level of actin isolation. Additionally, algorithms for analysis of the percentage of sliding filaments should be applied. However, the presented protocol provides tools to investigate effects of mutations or posttranslational modifications on the regulatory proteins, myosin heavy and light chains and actin.

Zebrafish skeletal muscle actin can be isolated and studied in interaction with different kinds of myosin; for example, when actin mutations are characterized, mutated zebrafish actin can be applied in a “standard IVMA” making use of HMM as the motor protein, which is also commercially available. Additionally, zebrafish actin can provide a basis for functional studies of the actin-myosin binding region as it was found to minimally differ in the amino acid sequence at the binding site compared to rabbit actin (Bertola et al., 2008). These small sequence differences could partly explain the variations of the sliding speed distributions we observed between zebrafish and rabbit actin on rabbit HMM (Figure 2D).

Actin isolation with prior acetone powder preparation is relatively time consuming, taking up to 3 days. Nevertheless, as the number of isolation steps was reduced in comparison to previously published methods (Pardee and Spudich, 1982; Kron et al., 1991), it is still an improvement regarding the involved effort. Although protein yield in this approach seems low (e.g., 6 mg actin from 350 mg acetone powder is less than 2%), a large number of single IVMA experiments can be carried out using the same actin source as less than 0.08 μg are needed in one experiment. We were also able to prepare acetone powder from tail muscles of individual zebrafish. This can provide a basis for actin analysis at the level of a single muscle tissue sample.

Currently, the view on data analysis of IVMA records has changed from usage of average sliding speeds to determining the fastest sliding filaments as these are supposed to move under optimal conditions (Aksel et al., 2015). In this context the myosin extraction protocol represents an optimized experimental setup providing images that simplify the accurate analysis of the maximum sliding velocity. Compared to conventional IVMA setups, the filament movement is relatively undisturbed and smooth (Figures 5B–D). If the SNR is high, segmentation-based automatic IVMA analysis methods can be used. However, automatic filament segmentation and the following velocity analysis is error-prone, if the SNR is low. In image sequences where the filaments move in a “stream”-formation, the background signal is often high due to the labeling of endogenous actin by excess Rhodamin-Phalloidin; in these cases, manual tracking should be used.

Taken together, we provide here the first protocol for zebrafish muscle protein extraction which can be used for IVMA. Cardiac myosin extracted from individual zebrafish ventricles is functional in IVMA and actin-myosin interaction can be studied also in Ca^2+^ dependence. Zebrafish IVMA can complement methods in zebrafish muscle research for analysis of Ca^2+^- regulation and contractile properties at the molecular functional level. Additionally, the presented myosin extraction protocol was shown to be applicable for muscle tissue of other origin, such as mouse skeletal and cardiac muscles, zebrafish skeletal and frog cardiac muscle. As functional myosin can be extracted within minutes after tissue dissection, this method can be used in studies of—besides others—posttranslational modifications as shown for ELC-phosphorylation (Scheid et al., 2016).

## Author contributions

LS, CW, and RF prepared the concept of this work; data acquisition was done by LS and CW; data were analyzed and interpreted by LS, CW, NB, MM, and RF. LS drafted the protocol, which was critically revised by all authors.

### Conflict of interest statement

The authors declare that the research was conducted in the absence of any commercial or financial relationships that could be construed as a potential conflict of interest.

## Abbreviations

AB: assay buffer
*ba*: *bulbous arteriosus*
BA: buffer A
CCD: charge-coupled device
EB: extraction buffer
EDL: *extensor digitorum longus*
ELC: essential myosin light chain
F-actin: filamentous actin
fps: frames per second
FV: frog ventricle
IVMA: *in vitro* motility assay
LB: labeling buffer
ME: mouse EDL
MV: mouse ventricle
RA: actin from rabbit skeletal muscle
(rab)HMM: heavy meromyosin (of rabbit origin)
RKH: Ringer-Krebs-Henseleit solution
RLC: regulatory myosin light chain
S1: (myosin) subfragment 1
SB: skinning buffer
SNR: signal-to-noise ratio
ZA: zebrafish skeletal muscle actin
ZFT: zebrafish tail muscle
ZFV: zebrafish ventricle.
