# Supplementary material for: Extraction Protocols for Individual Zebrafish's Ventricle Myosin and Skeletal Muscle Actin for In vitro Motility Assays
Source: Front Physiol. 2017 May 31;8:367. doi: 10.3389/fphys.2017.00367 (PMC5450195; doi:10.3389/fphys.2017.00367)
Supplement: Supplementary file 2 [file DataSheet1.docx]

Supplementary Material

Extraction Protocol for Individual Zebrafish’s Ventricle Myosin and Skeletal Muscle Actin for *In vitro* Motility Assays

Lisa-Mareike Scheid^1*^, Cornelia Weber^1^, Nasrin Bopp^1^, Matias Mosqueira^1^, Rainer H.A. Fink^1*^

^1^ Medical Biophysics Unit, Institute of Physiology and Pathophysiology, Medical Faculty, University of Heidelberg, Heidelberg, Germany

*** Correspondence:**Lisa-Mareike Scheid, Rainer H.A. Fink
lisa.scheid@physiologie.uni-heidelberg.de
rainer.fink@physiologie.uni-heidelberg.de

# Supplementary Data

A Supplementary table 1 listing all materials used for the described protocol is provided as an excel data sheet; all components are listed in the order of their first appearance in the protocol.

# Supplementary Figures and Tables

## Supplementary Figures


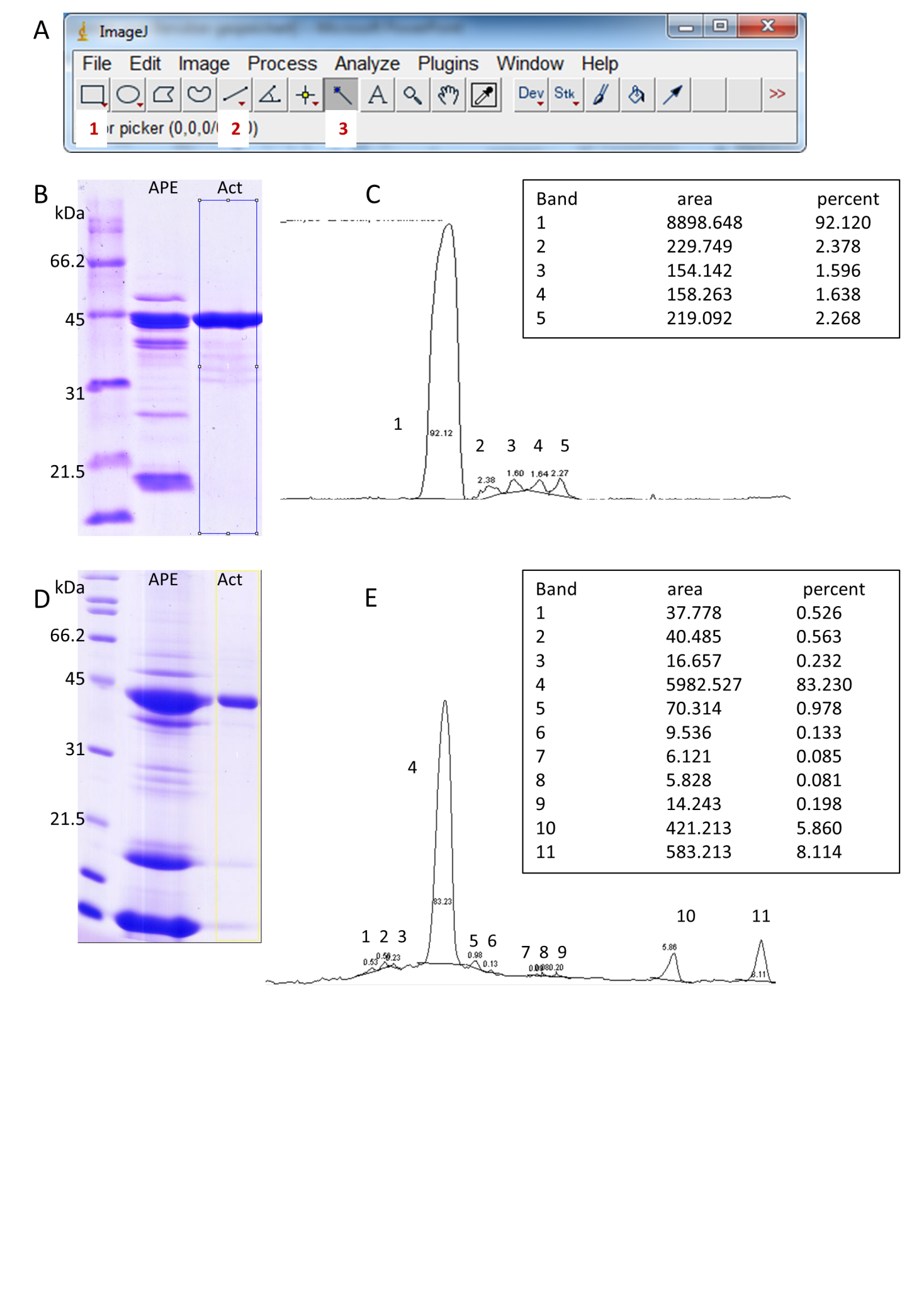
**Supplementary Figure 1.** **Use of ImageJ for protein quantification in SDS-PAGE scans**. For the Coomassie stained SDS-PAGE shown in Figure 2A (**B**) an additional batch of isolated zebrafish actin (**D**), protein band analysis is shown. APE stands for acetone powder extract and Act is for the isolated zebrafish actin. For analysis in ImageJ (**A**) the first lane of interest on the gel is framed with the “Rectangular” tool (**A**, 1), and chosen by CTR+1 (or “Analyze” – “Gels” – “Select first lane”); if more than one lane needs to be analyzed, the rectangular selection is moved to the second lane of interest that is chosen by CTR+2 (or “Analyze” – “Gels” – “Select next lane”) and so on. CTR+3 (or “Analyze” – “Gels” – “Plot lanes”) results in a plot of the intensity profile (**C, E**). With the “Straight” tool (**A**, 2) draw several lines to separate the intensity peaks from the background level. With the “Wand (tracing)” tool (**A**, 3) each peak is selected. “Analyze” – “Gels” – “label peaks” will indicate the area or percentage (when chosen in “Analyze” – “Gels” – “Gel analyzer options”) for each peak. ImageJ will automatically provide tables with the respective results (**C, E**).

**
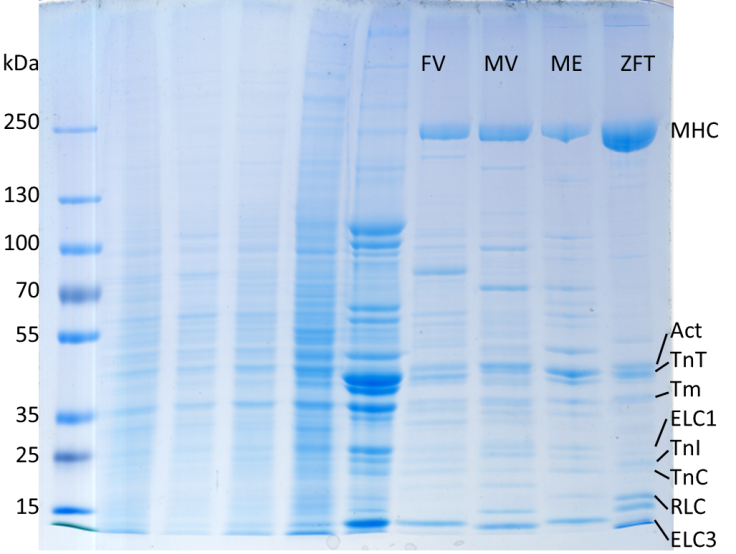
**

**Supplementary Figure 2. Image of the complete SDS-PAGE gel scan of Figure 3 B.** In Figure 3 B the protein standard (lane 1) is merged with the 4 lanes on the right side of the gel. Myosin extracts from frog ventricle (FV), mouse ventricle (MV), mouse EDL (ME) and zebrafish tail muscle (ZFT) are indicated. Myosin heavy chain (MHC), actin (Act), tropomyosin (Tm), troponin T (TnT), troponin I (TnI), troponin C (TnC), myosin essential light chain (ELC) and myosin regulatory light chain (RLC) protein bands are identified. In fast skeletal muscle two ELC isoforms are expressed (indicated as ELC1 and ELC3).
